# Inherent variability of cancer-specific aneuploidy generates metastases

**DOI:** 10.1186/s13039-016-0297-x

**Published:** 2016-12-16

**Authors:** Mathew Bloomfield, Peter Duesberg

**Affiliations:** 1 Department of Molecular and Cell Biology; Donner Laboratory, University of California at Berkeley, Berkeley, CA 94720 USA; 2 Present address: Department of Natural Sciences and Mathematics, Dominican University of California, San Rafael, CA USA

## Abstract

**Background:**

The genetic basis of metastasis is still unclear because metastases carry individual karyotypes and phenotypes, rather than consistent mutations, and are rare compared to conventional mutation. There is however correlative evidence that metastasis depends on cancer-specific aneuploidy, and that metastases are karyotypically related to parental cancers. Accordingly we propose that metastasis is a speciation event. This theory holds that cancer-specific aneuploidy varies the clonal karyotypes of cancers automatically by unbalancing thousands of genes, and that rare variants form new autonomous subspecies with metastatic or other non-parental phenotypes like drug-resistance – similar to conventional subspeciation.

**Results:**

To test this theory, we analyzed the karyotypic and morphological relationships between seven cancers and corresponding metastases. We found (1) that the cellular phenotypes of metastases were closely related to those of parental cancers, (2) that metastases shared 29 to 96% of their clonal karyotypic elements or aneusomies with the clonal karyotypes of parental cancers and (3) that, unexpectedly, the karyotypic complexity of metastases was very similar to that of the parental cancer. This suggests that metastases derive cancer-specific autonomy by conserving the overall complexity of the parental karyotype. We deduced from these results that cancers cause metastases by karyotypic variations and selection for rare metastatic subspecies. Further we asked whether metastases with multiple metastasis-specific aneusomies are assembled in one or multiple, sequential steps. Since (1) no stable karyotypic intermediates of metastases were observed in cancers here and previously by others, and (2) the karyotypic complexities of cancers are conserved in metastases, we concluded that metastases are generated from cancers in one step – like subspecies in conventional speciation.

**Conclusions:**

We conclude that the risk of cancers to metastasize is proportional to the degree of cancer-specific aneuploidy, because aneuploidy catalyzes the generation of subspecies, including metastases, at aneuploidy-dependent rates. Since speciation by random chromosomal rearrangements and selection is unpredictable, the theory that metastases are karyotypic subspecies of cancers also explains Foulds’ rules, which hold that the origins of metastases are “abrupt” and that their phenotypes are “unpredictable.”

## Background

Metastasis is defined as the development of secondary malignant growths at a distance from a primary site of cancer [1]. The relation of a metastasis to its primary cancer was described by Foulds in 1965 as a “progression” which, “is not a mere extension of a pre-existing lesion in space and time but a revolutionary change in a portion of the old lesion establishing a tumor with new properties not formerly present” [2]. Current textbooks point out that metastasis accounts for about 90% of the mortality from cancers and that, despite enormous efforts, its origins are still unclear [3, 4]. For example, *The Molecular Biology of the Cell* states that metastasis “is the most deadly – and least understood – aspect of cancer” [4].

### No evidence for consistent metastasis-specific mutations

In 1954 Foulds showed that cancers progress to metastases stochastically or “abruptly” and with diverse “unpredictable” phenotypes and concluded that “it is inadvisable to attribute to mutation” the great diversity of the individual phenotypes of metastases [5]. In apparent agreement with Foulds, no consistent metastasis-specific gene mutations [6–19], aneuploidies (abnormal chromosomes) [20–48] and transcriptomes [47, 49–64] have since been found. Based on a survey of influential studies on metastasis Michaelson et al. also concluded in 2005, “There has been much uncertainty as to whether metastasis requires mutation at the time of spread” [6, 65–73]. In view of this Michaelson et al. studied the probability of metastatic spread of clinical cancers and arrived at the conclusion that metastasis follows a “nongenetic mechanism”, typically at rates below 10^-8 per cancer cell [9, 10]. And *The Biology of Cancer* states in 2014–60 years after Foulds first questioned mutations - that “it is clear that the identities of many of the genes that are specifically involved in programming metastasis have been elusive.” [3]. In searching for these elusive metastasis-genes, it was also observed that the proclivity of cancers to metastasize is determined prior to metastasis, is “preordained” [69] or “predetermined” [74]. Considering the elusive search for metastasis-genes and our prior work on the karyotypic basis of cancer and metastasis [43, 75–77] we have asked here, whether the proclivity of cancers to metastasize might be determined by cancer-specific aneuploidy.

### Two links between aneuploidy and metastasis

In an effort to find a mutation-independent mechanism of metastasis, we reviewed previously unexplained findings for clues. As a result we found two conspicuous links: (1) the risk of metastasis correlates with the degrees of cancer-specific aneuploidy, and (2) the aneuploid karyotypes of metastases are related to those of primary cancers. In the following we first summarize the evidence for these two links between aneuploidy and metastasis, and then show that a karyotypic, rather than a mutational theory can explain the origins of metastasis.

#### Correlations between degrees of cancer-specific aneuploidy and proclivity for metastasis

Studying cancer cytogenetics Atkin found in 1972 that “Carcinomas of the breast fell into two discrete groups. The lower near-diploid group showed a significantly better eight year survival rate than the higher triploid-tetraploid group.” [78]. Likewise, Frankfurt et al. observed in 1985, “Only 7.1% of diploid tumors with a Gleason score of 5 to 6 formed metastases, but 80% of aneuploid tumors with a higher Gleason score (7 to 10) formed metastases”, and concluded, “DNA ploidy may be an important prognostic factor for human prostate cancer.” [79]. In a review on “tumor progression” Wolman wrote in 1986, “There was a significantly higher survival rate and lower metastasis frequency associated with diploid tumors. A large majority (81%) of the tumors which metastasized were hyperploid.” [80]. The same conclusion was reached in 1986 by Ljungberg et al., who found that “deoxyribonucleic acid content might be a useful prognostic discriminator with implications for the clinical management of patients with metastatic renal cell carcinoma” [81]. Fallenius et al. made the same observation with breast cancers in 1988, “The diploid carcinomas represent slowly growing tumors with low risk of producing metastases and consequently, a highly favorable prognosis. In contrast, aneuploid tumors are potentially highly malignant variants, mostly rapidly progressing tumors with poor prognoses” [82]. Further, Saito et al. reported in 1994, “the incidence of the cervical lymph node metastasis was significantly (*P* < 0.02) higher in the aneuploid cases (8/15) than in the diploid cases (3/21)” [83]. In 1997 Hemmer et al. reached the same conclusion based on DNA content in a study entitled, “The value of DNA flow cytometry in predicting the development of lymph node metastasis and survival in patients with locally recurrent oral squamous cell carcinoma” [84]. And Torres et al. observed in 2007 that, “the number of genomic imbalances in primary (breast) tumors was significantly higher in patients presenting lymph node metastases (median = 15.5) than in the group with no evidence” for metastasis [85]. Concurrently Jonkers et al. reached the conclusion in 2007 that “DNA copy number status is the most sensitive and efficient marker of adverse clinical outcome (of pancreatic cancers); particularly of metastatic disease” [41], which was confirmed by an independent study from Norway in 2014 [44].

Despite these consistent correlations between degrees of aneuploidy and metastasis, the functional role of aneuploidy in metastasis remained unexplained [3, 4, 86, 87].

#### Karyotypic relationships between the aneuploidies of cancers and their metastases

Numerous independent studies found that the individual aneuploid karyotypes of metastases are related to those of individual primary cancers, but not to those of metastases of other cancers [20–46, 75, 88]. Metastases also have individual transcriptomes and gene mutations that are exclusively related to those of parental cancers [3, 11–18, 49–64, 89]. The 1-to-1 correlations between the individual karyotypes and individual transcriptomes of metastases indicate that these individual transcriptomes encode the individual phenotypes of metastases.

But in view of the individuality of the karyotypes and transcriptomes of metastases, compared to the expected common metastasis-specific mutations, a coherent theory of the role of the individual karyotypes and transcriptomes of metastases has not emerged. Therefore, we test here the theory that metastasis is a form of speciation, which predicts individual metastasis-specific karyotypes and phenotypes, rather than common mutations. This theory extends and confirms preliminary karyotypic evidence for metastasis from two labs including ours [43, 75, 88].

### Karyotypic origins of metastasis

**Fig. 1:**
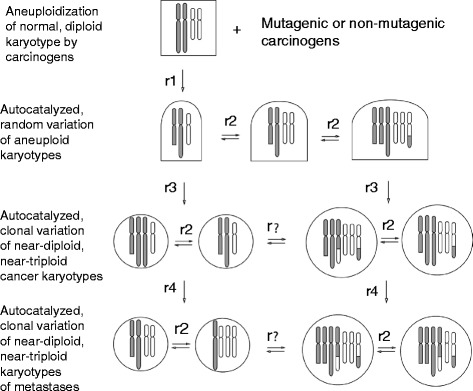
Karyotypic theory of metastasis. According to the karyotypic theory mutagenic and non-mutagenic carcinogens induce random aneuploidy in normal cells at carcinogen-dependent rates, r1. Aneuploidy then auto-catalyzes further random karyotypic variation, because it unbalances thousands of genes including mitosis-genes at aneuploidy-dependent rates, r2. Most such random variants perish. Rare karyotypic variants, however, form new autonomous cancer species with near-diploid, hypo-diploid or hyper-diploid karyotypes at very low rates, r3, because the probability to form a new autonomous cell is very low [75–77, 104, 105, 109, 135]. Owing to the inherent instability of aneuploidy, the karyotypes of new cancer species vary at aneuploidy-dependent rates, r2, within margins that are defined by selection for cancer-specific autonomy. As a result of this inherent variability of cancer karyotypes, new autonomous karyotypic subspecies with metastatic phenotypes arise stochastically. Since the probability of forming new autonomous subspecies by random variation of cancer karyotypes is very low – as in conventional subspeciation – metastases would occur typically at low rates, r4, such as the 10^-8 per-cell rate described in the Background in reference [9]. These rates are still higher than those of carcinogenesis from normal cells, r3 [136]. The Figure also records graphically our results that the karyotypic complexity of cancers is highly conserved in metastases. Thus hyper-triploid cancers formed hyper-triploid metastases and near-diploid cancers formed near-diploid metastases. There was no evidence of spontaneous alterations of the clonal karyotypic complexity of cancers or metastases; hence ‘r?’ in Fig. 1

The theory that metastases are karyotypic subspecies of cancers is a logical extension of the theory that cancers are karyotypic species of their own [75, 90, 91]. According to this theory metastasis is the end product of a sequence of karyotypic variations (Fig. 1). This sequence of variations starts with the induction of random aneuploidy in normal cells (squares in Fig. 1) by carcinogens. Aneuploidy then catalyzes further random karyotypic variation of aneuploid cells (half-squares in Fig. 1) automatically, because aneuploidy unbalances thousands of genes including mitosis-genes, at aneuploidy-dependent rates (r2, Fig. 1). Most such random variants perish. Stochastically, very rare karyotypic variants of aneuploid cells form new autonomous cancer cells (circles in Fig. 1) at very low, aneuploidy-dependent rates (r3, Fig. 1). Owing to the inherent instability of cancer-specific aneuploidy cancer karyotypes vary within margins defined by selection for cancer-specific autonomy, again at aneuploidy-dependent rates (r2, Fig. 1). Eventually, very rare new autonomous variants or new subspecies arise from cancers with metastatic or other new phenotypes, like drug-resistance at low aneuploidy-dependent rates (r4, Fig. 1).

One may argue, however, that cancers are not species, because they lack a natural habitat beyond their original host. The American evolutionary biologist Lee Van Valen acknowledged this reservation, because “cancer cells depend for their existence on the continuation of the practice of tissue culture”. But Van Valen maintained that this “wholely artificial” habitat is not an argument against the species definition of cancers, “the expected persistence of its habitat is never used as a criterion for judging the reality of a species, but rather for judging its susceptibility to extinction” [92]. Moreover, unbeknown to Van Valen several cancers have found niches for infinite natural transmissions without natural graft resistance in dogs [93], Tasmanian devils [88, 93] and clams [94]. Thus cancers are species, although their natural habitats are restricted.

In the following we have tested the theory that metastases are subspecies of cancers by comparing the karyotypes of seven independent cancers with corresponding metastases.

## Results and discussion

### Karyotypic and phenotypic relationships between metastases and parental cancers

As a first test of our theory that metastases are subspecies of cancers, we analyzed the karyotypic and cellular phenotypic relationships between seven published pairs of cancers and metastases:

1. The breast cancer, HIM-2 and a brain metastasis, HIM-5 [16].
2. The melanoma, WM-115 and a metastasis of undefined origin, WM-266-4 [21–23]. It is demonstrated in Fig. 3 that both cancers analyzed here derived from an unknown common precursor.
3. The liver cancer, H2P and a metastasis in the portal vein to the liver, H2M [35].
4. The medulloblastoma, M-458 and a metastasis from a cerebrospinal fluid relapse, M-425 [95, 96]. A comparison between the M-458 and M-425 received in 2014 for this study with those published previously by Bigner et al. in 1991 revealed a discrepancy in chromosome copy numbers, although marker chromosomes were conserved. As a result Dr. Bigner left it up to us to decide, which variant was the original cancer or metastasis. Based on our results, e.g. the higher growth rate, growth in suspension and higher clonality of M-425 compared to M-458 described below, we named M-425 the metastasis.
5. The colon cancer, SW-480 and a lymph node metastasis, SW-620 [97–101] with evidence, shown below, that both are derivatives of an unknown common precursor.
6. The melanoma, IGR-39 from a leg and a metastasis from the groin, IGR-37 [102].
7. The pancreatic cancer, A13-B and two independent metastases, a pancreatic metastasis A13-A and a liver metastasis A13-D [43].

The provenance of these cancers and corresponding metastases, and the conditions under which they were propagated are described in Methods.

The theory that metastases are subspecies of cancers makes two testable predictions: (1) Each pair of a cancer and corresponding metastasis is karyotypically related. (2) Each pair is also distinct in cancer- and metastasis-specific karyotypic and phenotypic markers. To test these predictions we compared the karyotypes and morphological cellular phenotypes of each of these seven cancer-metastasis pairs.

**Fig. 2:**
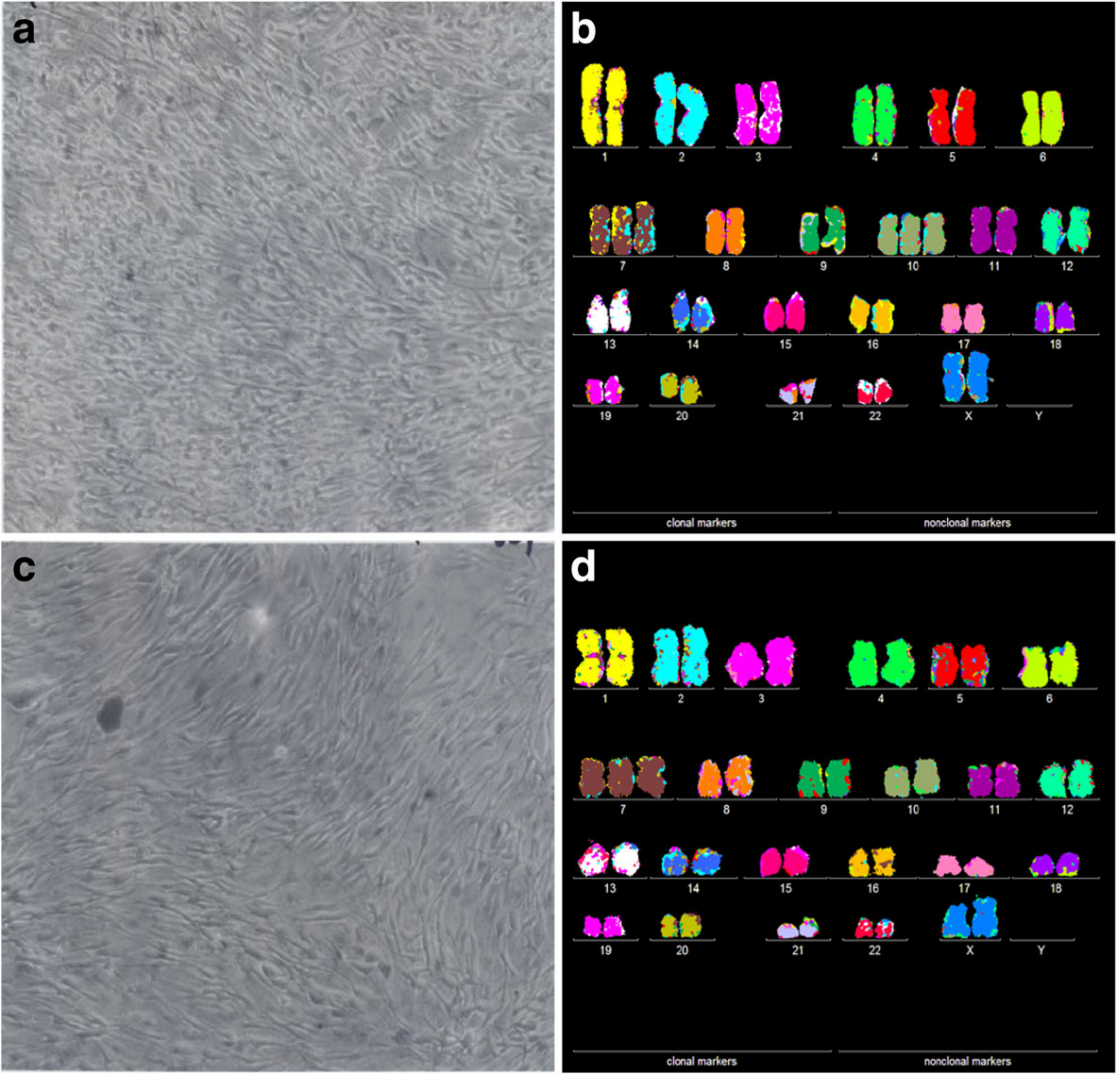
Cellular morphologies and karyotypes of breast cancer HIM-2 (**a**, **b**) and of a corresponding brain metastasis HIM-5 (**c**, **d**). The comparisons show that the primary cancer HIM-2 and corresponding metastasis HIM-5 have very similar but distinct cell morphologies (**a**, **c**) and karyotypes (**b**, **d**). Both primary cancer and metastasis have near diploid karyotypes with the same numbers and structures of chromosomes, except for a trisomy 10 that is missing in the metastasis HIM-5 (**d**). The cells were photographed at 120X in cell culture dishes (Methods). The cells of the primary cancer were found to be more refractive and growing at modestly higher rates than the metastasis. The chromosomes were prepared from metaphase-cells and color-coded as described in Methods

**Fig. 3:**
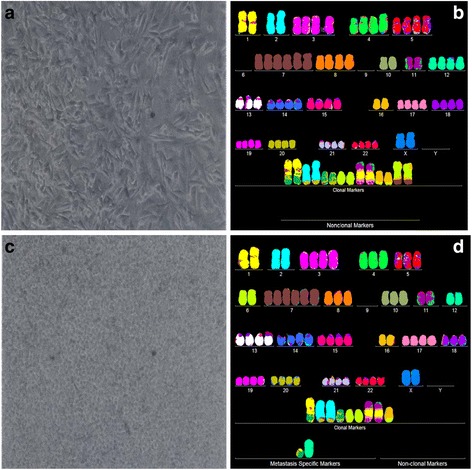
Cellular morphologies and karyotypes of melanoma WM-115 (**a**, **b**) and a corresponding metastasis WM-266-4 (**c**, **d**). The comparisons show that the primary cancer WM-115 and the corresponding metastasis WM-266-4 have similar, but distinct cell morphologies (**a**, **c**) and karyotypes (**b**, **d**). Both primary cancer and metastasis have hyper-triploid karyotypes with similar numbers of chromosomes and aneusomies and both lack intact chromosome 9. They also differ from each other in the total numbers of chromosomes and in the structures of some marker chromosomes (see Tables 1 and 2). The absence of normal chromosomes 6 from the presumed primary and the presence of "primary specific" marker chromosomes 6 indicate that both the presumed primary and the metastasis derived from an unknown primary with normal chromosomes 6. The cells were propagated and karyotyped as described for Fig. 2

**Fig. 4:**
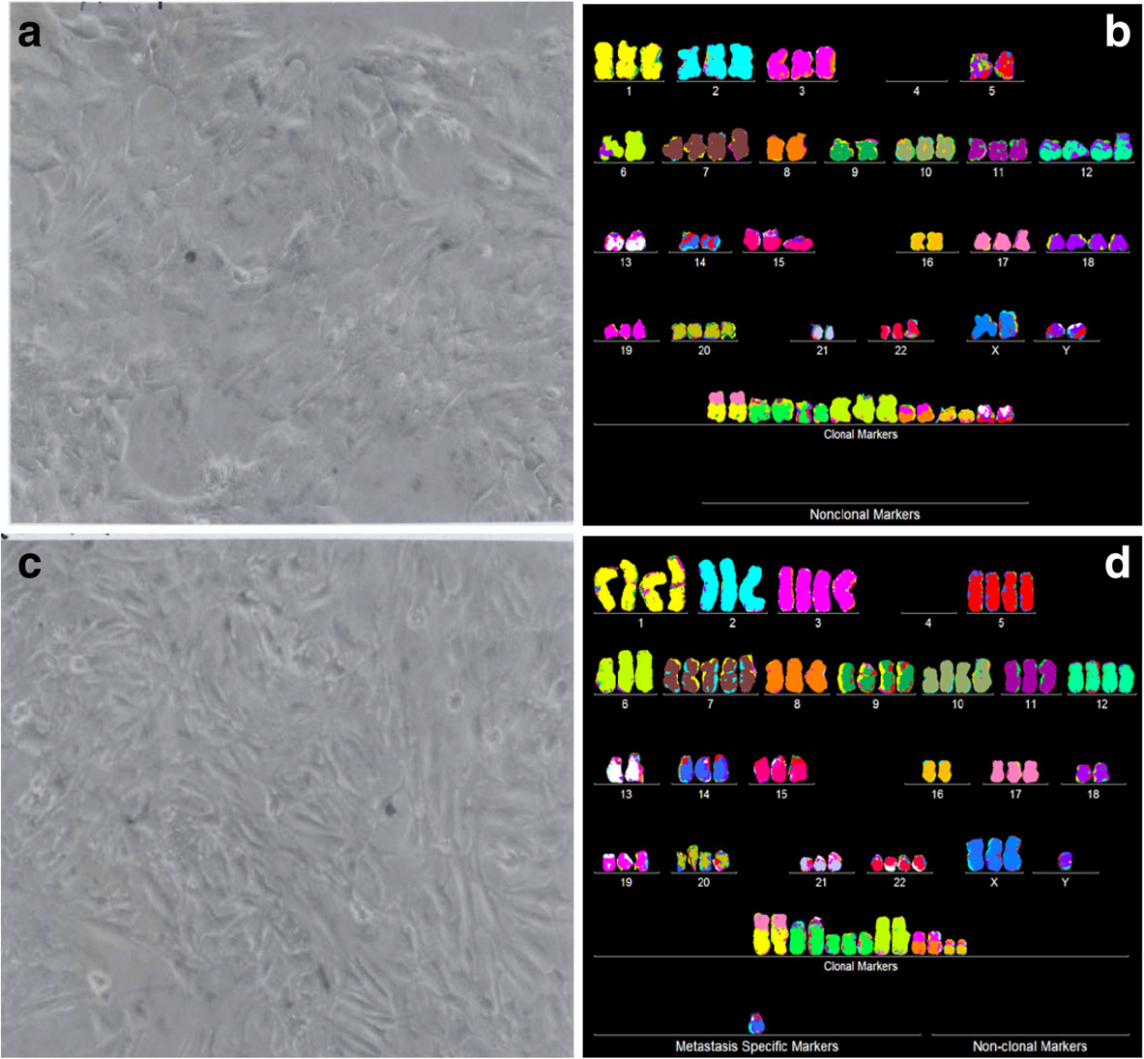
Cellular morphologies and karyotypes of liver cancer H2P (**a**, **b**) and a corresponding portal vein metastasis H2M (**c**, **d**). The comparisons show that the primary cancer H2P and the corresponding metastasis H2M have similar, but distinct cell morphologies (**a**, **c**) and karyotypes (**b**, **d**). Both primary cancer and metastasis have hyper-triploid karyotypes with similar numbers of chromosomes and aneusomies, and both lack intact chromosome 4. They differ from each other in the total numbers of chromosomes and in the structures of some marker chromosomes (see Tables 1 and 2). The cells were propagated and karyotyped as described for Fig. 2

**Fig. 5:**
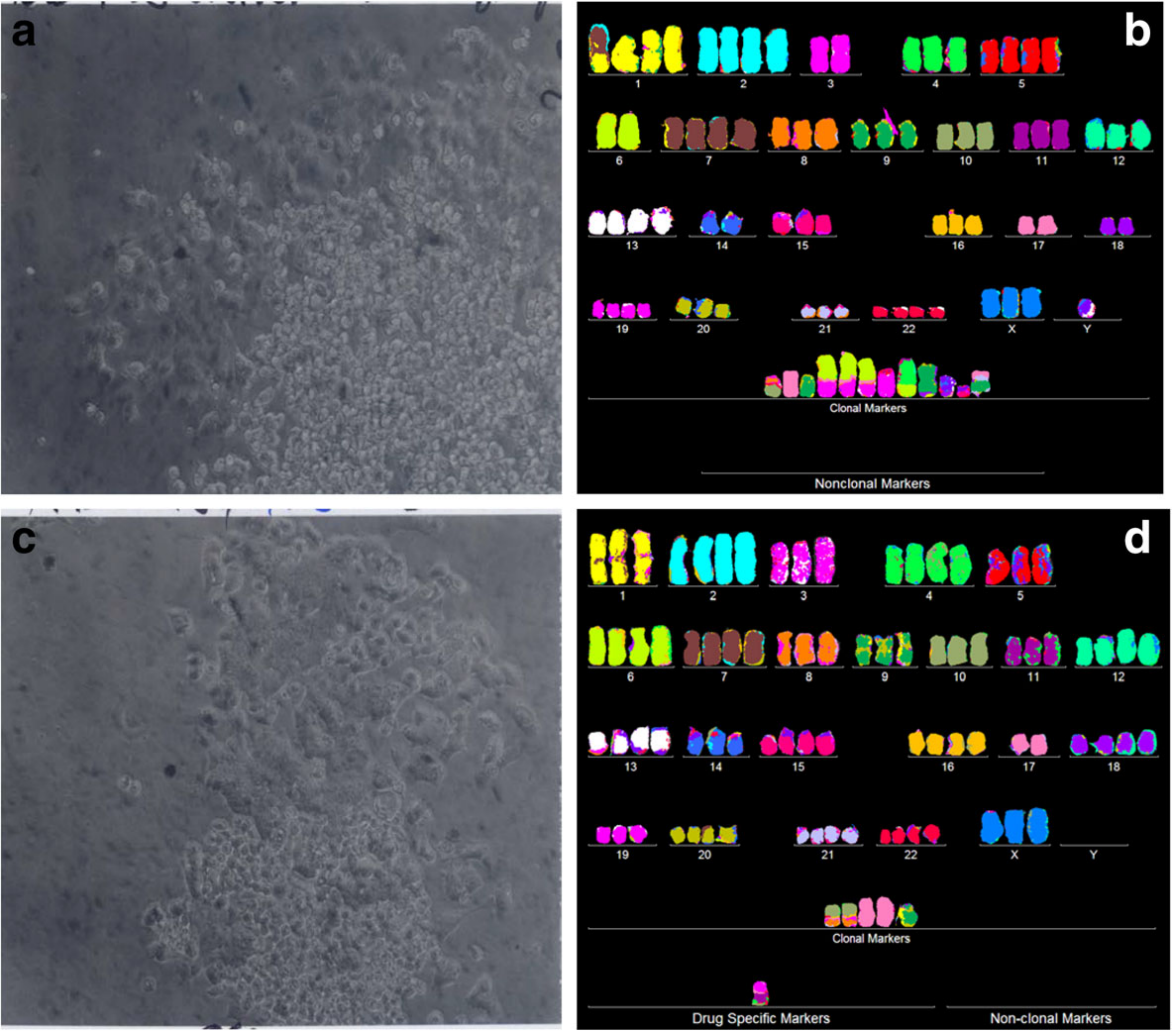
Cellular morphologies and karyotypes of medulloblastoma M-458 (**a**, **b**) and a corresponding metastasis M-425 (**c**, **d**). The comparisons show again that the primary cancer M-458 and the corresponding metastasis M-425 have similar, but distinct cell morphologies (**a**, **c**) and karyotypes (**b**, **d**). Some of the metastatic M-425 cells grew in suspension (One reason, why M-425 was named the metatasis. See note above in this section.), while the rest was attached to the culture dish. The karyotypes of both the primary cancer and the metastasis are hyper-triploid and have similar numbers of chromosomes and of aneusomies. But they also differ in the total numbers of chromosomes and in the structures of some individual marker chromosomes (see Tables 1 and 2). The cells were propagated and karyotyped as described for Fig. 2

**Fig. 6:**
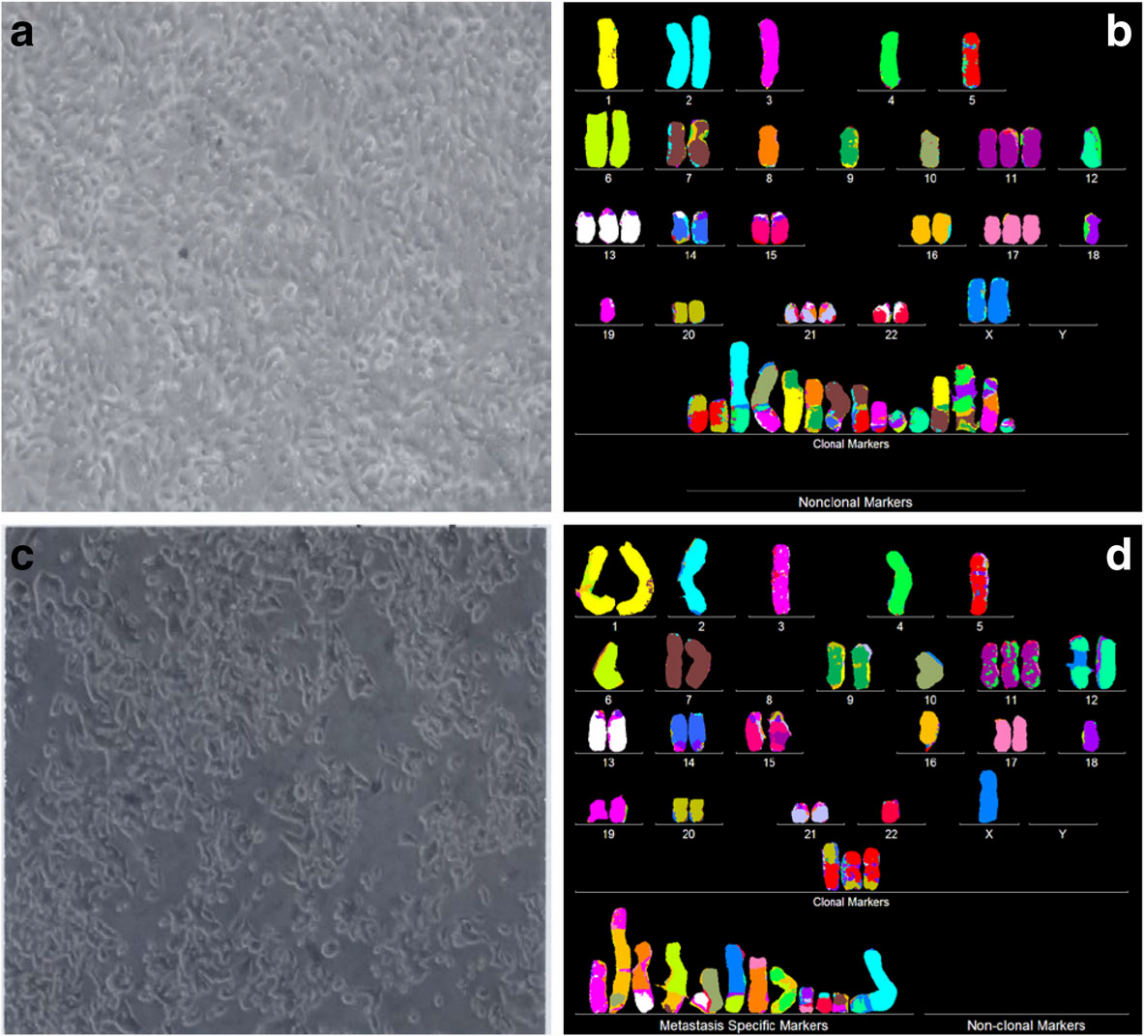
Cellular morphologies and karyotypes of colon cancer SW-480 (**a**, **b**) and of a corresponding metastasis SW-620 (**c**, **d**). The comparisons show once more that the primary cancer SW-480 and the corresponding metastasis SW-620 have similar, but distinct cell morphologies (**a**, **c**) and karyotypes (**b**, **d**). Both primary cancer and metastasis have hyper-diploid karyotypes with similar numbers of chromosomes and of aneusomies. They also differ from each other in the total numbers of chromosomes and in the structures of some marker chromosomes (see Tables 1 and 2). We adduce evidence in the text that both SW-480 and SW-620 are probably both metastases of an unknown primary. The cells were propagated and karyotyped as described for Fig. 2

**Fig. 7:**
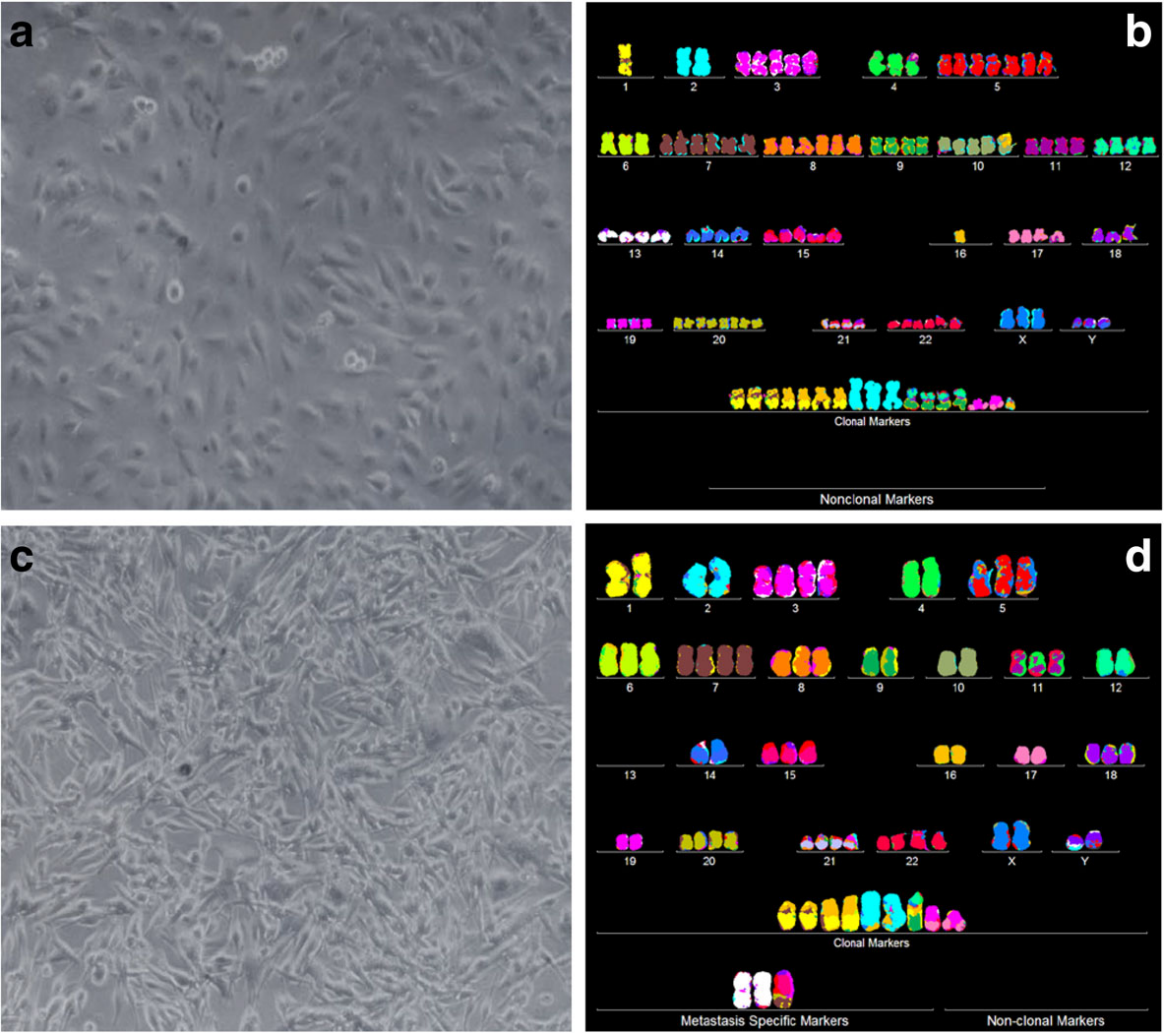
Cellular morphologies and karyotypes of melanoma IGR-39 (**a**, **b**) and of a corresponding metastasis IGR-37 (**c**, **d**). The comparisons show once more that the primary melanoma IGR-39 and the corresponding metastasis IGR-37 have similar, but distinct cell morphologies (**a**, **c**) and that their karyotypes are closely related but differ in ploidy number (**b**, **d**). The primary cancer has a hyper-tetraploid karyotype and the metastasis a closely related, but hyper-triploid karyotype. The relationship is based on chromosome copy numbers that differ from each other by the ploidy factor that sets apart the two karyotypes. The karyotypes also differ from each other in several individual chromosome numbers and in several individual marker chromosomes (see Tables 1 and 2). The cells were propagated and karyotyped as described for Fig. 2 and in the text

**Fig. 8:**
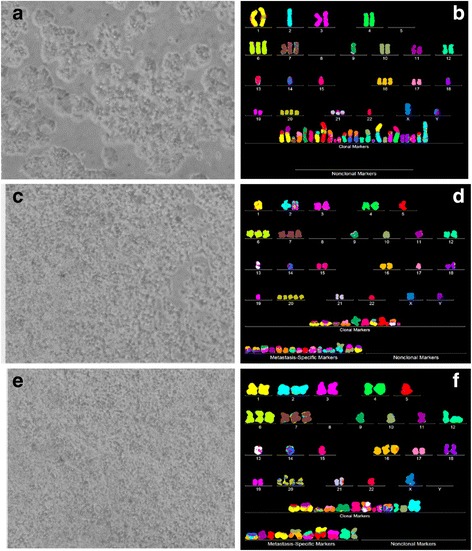
Cellular morphologies and karyotypes of pancreatic cancer A13-B (**a**, **b**) and of two corresponding metastases, a pancreatic metastasis A13-A (**c**, **d**) and a liver metastasis A13-D (**e**, **f**). The comparisons show once more that the primary cancer A13-B and the corresponding metastases A13-A and A13-D have similar, but distinct cell morphologies (**a**, **c**, **e**) and karyotypes (**b**, **d**, **f**). The primary cancer and both metastases have hyper-diploid karyotypes with similar numbers of chromosomes and of aneusomies. The primary and the two metastases differ from each other in the total numbers of chromosomes and in the structures of some individual marker chromosomes see Tables 1 and 2). The cells were propagated and karyotyped as described for Fig. 2 [103]

As can be seen in Figs. 2, 3, 4, 5, 6, 7, 8, the results of these tests revealed the following similarities and dissimilarities between cancers and corresponding metastases:

1. The microscopic cellular phenotypes of all seven cancers were similar to, but also distinct from those of the corresponding metastases. In addition we found non-microscopic distinctions that set apart cancers from metastases. For example, the cellular growth rate of the primary melanoma WM-115, shown in Fig. 3, was about five-fold lower than that of the metastasis WM-266-4. Likewise the growth rate of the primary melanoma IGR-37 was lower than that of the corresponding metastasis IGR-39 (Fig. 7). The primary medulloblastoma M-458 also differed from the corresponding metastasis M-425 in two physiological characteristics: The M-458 cancer cells grew slower than those of the metastasis M-425; and the M-458 cells were mostly attached to the culture dish, whereas the cells of the metastasis grew mostly in suspension, hence out of focus in Fig. 5.
2. The numbers of chromosomes of all seven cancers were very similar to, but not identical to those of the corresponding metastases.
3. The karyotypes of all seven cancers shared a majority of structurally and numerically-defined aneusomies with corresponding metastases. But they also all differed from each other in distinct cancer- and metastasis-specific aneusomies.
4. The phenotypic and karyotypic differences between cancers and metastases were roughly proportional to the degrees of cancer-specific of aneuploidy: The more aneuploid the cancer, the more different are the karyotypes of cancers from those of corresponding metastases. For example, it is shown Fig. 2 that the near-diploid breast cancer HIM-2 differed from the brain metastasis HIM-5 only in the loss of one of two trisomies, namely trisomy 10. By contrast the six near-triploid cancers shown in Figs. 3, 4, 5, 6, 7, 8, differed from the corresponding metastases in high percentages of their constituent aneusomies (see also below).

We deduce from these comparative analyses that the karyotypes and phenotypes of metastases are exclusively related to, but also distinct from parental cancers – exactly as predicted by the theory that metastases are subspecies of cancers. Our observations that metastases appear to conserve the average numbers of parental chromosomes and conserve many parental aneusomies further supports the subspeciation theory – as conventional species typically conserve the genetic complexities and most of the karyotypes of their precursors [103].

It could be argued, however, that the differences setting apart the karyotypes of metastasis from parental cancers shown in Figs. 2, 3, 4, 5, 6, 7, 8 are random karyotypic variants generated by the inherent instability of cancer-specific aneuploidy, and that as yet un-identified gene mutations generate metastases from cancers. If that were correct, the karyotypes of metastases would not be stabilized by selection for autonomy and thus would not be clonal, and the numbers of their chromosomes would not, or not consistently be close to those of parental cancers.

By contrast, if metastases were karyotypic subspecies of cancers, their karyotypes would be clonally related to parental cancers and would be stabilized by selection for autonomy - like those of parental cancers and of conventional species.

To distinguish between these possibilities we next have investigated, whether the karyotypes of metastases are clonal and are clonally related to parental cancers.

### Metastases are karyotypic subspecies of cancers

To prove that cancers cause metastases by karyotypic variation rather than by gene mutation, we need evidence that the karyotypes of metastases are clonal and clonally related to parental karyotypes.

To determine karyotypic clonality, the karyotypes of multiple cells of a metastasis and of a primary cancer must be compared and shown to be identical. But, owing to the inherent variability or flexibility of cancer karyotypes and the resulting clonal heterogeneity of cancers [75, 88, 104, 105] (Background), the determination of clonality of karyotypes is often obscured in conventional karyotypic analyses of cancers by clonal heterogeneity [7, 106, 107].

**Fig. 9:**
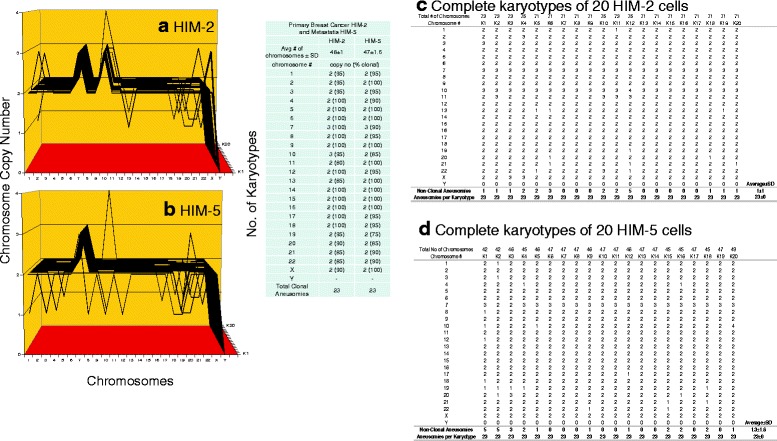
**a**, **b**, **c**, **d** Karyotypic evidence that the brain metastasis HIM-5 is an individual subspecies of the breast cancer HIM-2. The karyotypic theory of metastasis predicts that metastases have individual clonal karyotypes that differ from those of parental cancers in individual metastasis-specific aneusomies. To test this theory we have compared karyotype-arrays of the brain metastasis HIM-5 to that of the primary cancer HIM-2. Karyotype arrays are three-dimensional tables of 20 karyotypes, which list the chromosome numbers of arrayed karyotypes on the x-axis, the copy numbers of each chromosome on the y-axis, and the number of karyotypes arrayed on the z-axis, as detailed in Results (Section, Metastases are karyotypic subspecies of cancers). Figure 9 **a**, **b** and the attached table show that 85 to 100% of the chromosomes of the cancer HIM-2 and the metastasis HIM-5 were clonal, and that cancer and metastasis formed very similar clonal patterns. The karyotype of the metastasis differed from that of the primary only in the loss of trisomy 10. The copy numbers of the non-clonal chromosomes differed from the clonal averages typically  ± 1 (see Fig. 9 **a**,**b** and specifically Fig. 9 **c**,**d**). These non-clonal copy numbers represent the ongoing karyotypic variation predicted by the inherent variability of cancer-specific aneuploidy (Background). We conclude that the brain metastasis HIM-5 is a subspecies of the parental breast cancer HIM-2

**Fig. 10:**
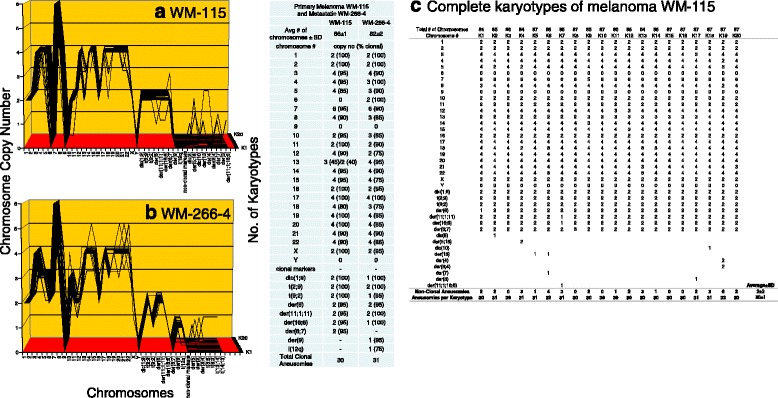
**a**, **b**, **c** Karyotypic evidence that the metastasis WM-266-4 is an individual subspecies of melanoma WM-115. The karyotypic theory of metastasis predicts that metastases have individual clonal karyotypes that differ from those of parental cancers in individual metastasis-specific aneusomies. To test this theory we have compared karyotype-arrays of the melanoma metastasis WM-266-4 to that of the primary cancer WM-115 prepared as described for Fig. 9. Figure 10 **a**, **b** and the attached table show that 80 to 100% of the chromosomes of the metastasis WM-266-4 and of the cancer WM-115 were clonal, and that cancer and metastasis both formed very similar clonal patterns. The karyotype of the metastasis differed from that of the primary cancer in about 13 of an average of 31 aneusomies (Fig. 10 **a**, **b**, **c** and Table 1). The copy numbers of the non-clonal chromosomes differed from clonal averages ± 1; there were also several non-clonal marker chromosomes (Fig. 10 **a**, **b**, **c**). These non-clonal chromosomes represent the ongoing karyotypic variation predicted by the inherent variability of cancer-specific aneuploidy (see Fig. 10 **a**, **b** and specifically Fig. 10**c**, and Background). We conclude that the melanoma metastasis WM-266-4 is a subspecies of the parental melanoma WM-115

**Fig. 11:**
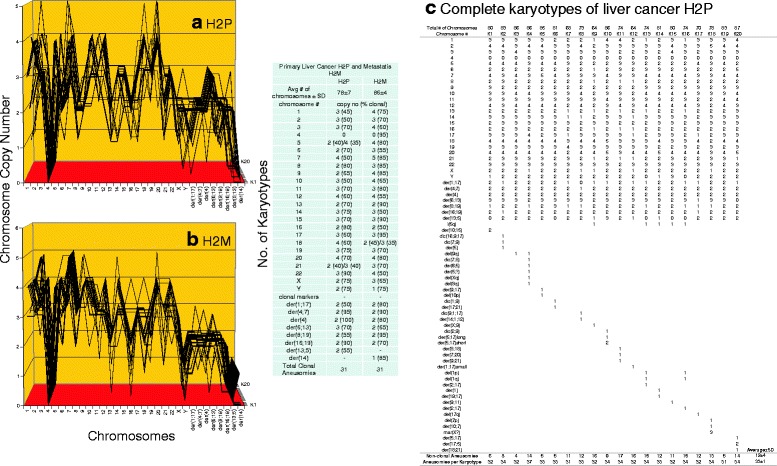
**a**, **b**, **c** Karyotypic evidence that the metastasis H2M is an individual subspecies of liver cancer H2P. The karyotypic theory of metastasis predicts that metastases have individual clonal karyotypes that differ from those of parental cancers in individual metastasis-specific aneusomies. To test this theory we have compared karyotype-arrays of the metastasis H2M to that of the primary cancer H2P prepared as described for Fig. 9. Figure 11 **a**, **b** and the attached table show that 45–80% of the chromosomes of cancer H2P and 50–90% of the chromosomes of metastasis H2M were clonal, and that cancer and metastasis formed similar clonal patterns. The karyotype of the metastasis differed from that of the primary cancer in about 15 of an average of 31 H2M aneusomies (Fig. 11 **a**, **b**, **c** and Table 1). The copy numbers of non-clonal chromosomes including marker chromosomes differed from clonal averages ± 1 (see Fig. 11 **a**, **b** and specifically Fig. 11**c**). The chromosomes with non-clonal copy numbers represent the ongoing karyotypic variation predicted by the inherent variability of cancer-specific aneuploidy (See Fig. 11**c** and Background). We conclude that the metastasis H2M is a subspecies of the parental liver cancer H2P

**Fig. 12:**
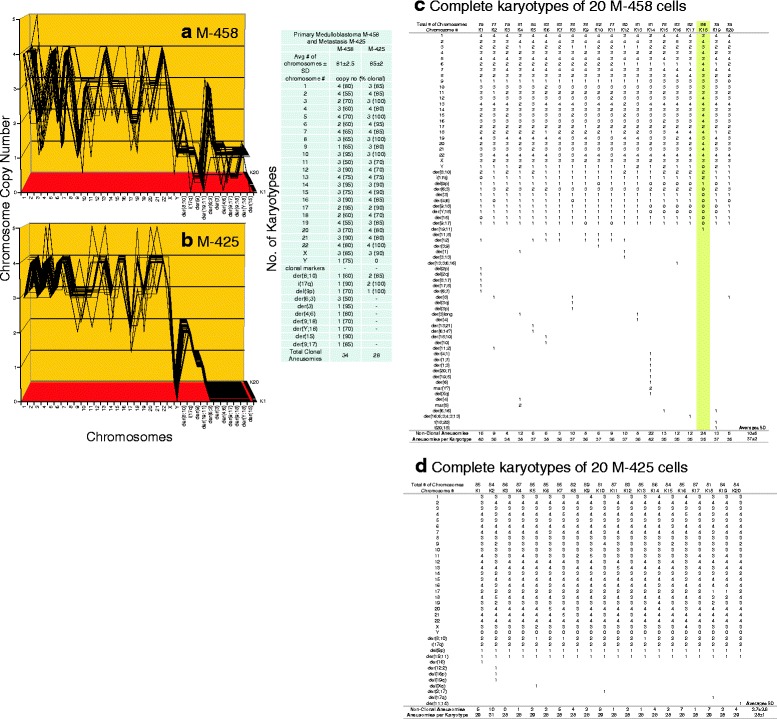
**a**, **b**, **c**, **d** Karyotypic evidence that the metastasis M-425 is an individual subspecies of medulloblastoma M-458. The karyotypic theory of metastasis predicts that metastases have individual clonal karyotypes that differ from those of parental cancers in individual metastasis-specific aneusomies. To test this theory we have compared karyotype-arrays of the metastasis M-425 to that of the primary cancer M-458 prepared as described for Fig. 9. Figure 12 **a**, **b** and the attached table show that 55–90% of the chromosomes of the cancer M-458 and 70–100% of the chromosomes of the metastasis M-425 were clonal, and that cancer and metastasis formed similar clonal patterns. The fact that the karyotype of M-425 was more clonal than that of M-458, again supports the view that M-425 is the metastasis and M-458 the original cancer (See comment regarding this question in section "Karyotypic and phenotypic relationships between metastases and parental cancers"). The karyotype of the metastasis differed from that of the primary cancer in about 17 of an average of 28 M-425 aneusomies (Fig. 12 **a**, **b**, **c**, **d** and Table 1). The copy numbers of most non-clonal chromosomes including marker chromosomes differed from clonal averages ± 1 (see Fig. 12 **a**, **b** and specifically Fig. 12 **c**, **d**). The chromosomes with non-clonal copy numbers represent the ongoing karyotypic variation predicted by the inherent variability of cancer-specific aneuploidy (See Fig. 11**c** and Background). We conclude that the metastasis M-425 is subspecies of the parental medulloblastoma M-458

**Fig. 13:**
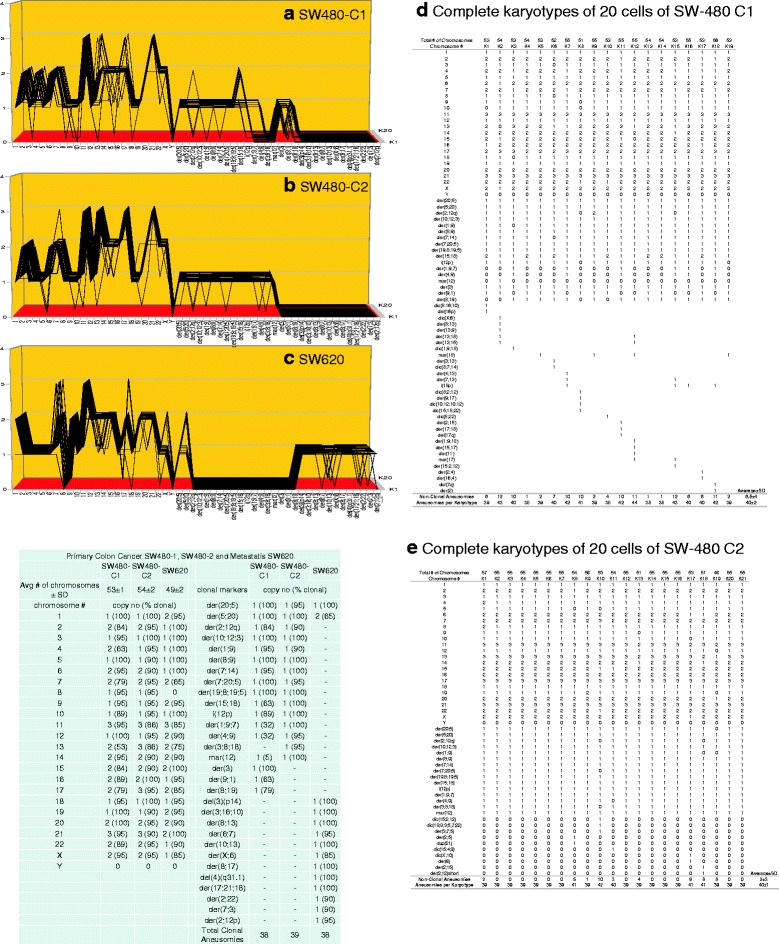
**a**, **b**, **c**, **d**, **e** Karyotypic evidence that the metastasis SW-620 is an individual subspecies of SW-480 or of an unknown common precursor. The karyotypic theory of metastasis predicts that metastases have individual clonal karyotypes that differ from those of parental cancers in individual metastasis-specific aneusomies. To test this theory we have compared karyotype-arrays of the metastasis SW-620 to that of two clones of the presumed primary SW-480, prepared as described for Fig. 9. Figure 13 **a**, **b**, **c** and the attached table show that 75 - 100% of the chromosomes of SW-620 and 53–100% of the chromosomes of SW-480 C1 and 75-100% of the chromosomes of SW-480 C2 were clonal, and that the two cancer clones and the metastasis formed similar clonal patterns. The karyotype of the metastasis differed from that of the primary cancer SW-480 C1 in 25 of 38 average SW-480 C1 aneusomies and differed from SW-480 C2 in 27 of 38 average aneusomies (Fig. 13**a**, **b**, **c**, **d**, **e** and Table 1). The copy numbers of most non-clonal chromosomes including marker chromosomes differed from clonal averages ± 1 (Fig. 13**a**, **b**, **c** and specifically Fig. 13**d**, **e**). The chromosomes with non-clonal copy numbers represent the ongoing karyotypic variation predicted by the inherent variability of cancer-specific aneuploidy (See Fig. 11**d**, **e** and Background). We conclude that the metastasis SW-620 is a subspecies of the parental colon cancer SW-480 or of a common unknown precursor

**Fig. 14:**
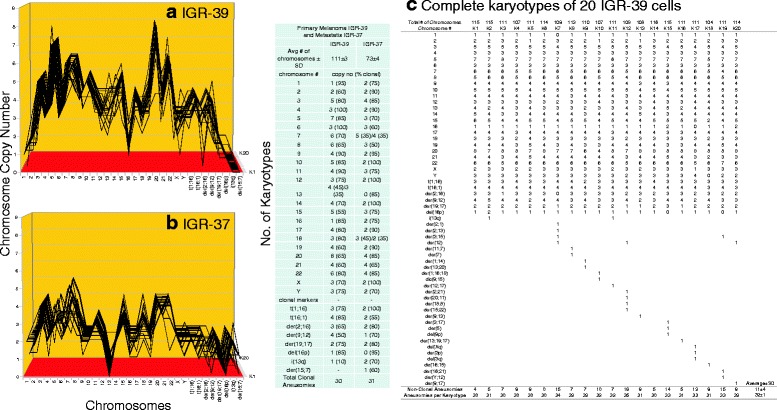
**a**, **b**, **c** Karyotypic evidence that the metastasis IGR-37 is an individual subspecies of melanoma IGR-39. The karyotypic theory of metastasis predicts that metastases have individual clonal karyotypes that differ from those of parental cancers in individual metastasis-specific aneusomies. To test this theory we have compared karyotype-arrays of the metastasis IGR-37 to that of the primary cancer IGR-39 prepared as described for Fig. 9. Figure 14 **a**, **b**, **c** and the attached table show that 55–100% of the chromosomes of the parental cancer IGR-39 and 50–100% of the chromosomes of the metastasis IGR-37 were clonal, and that cancer and metastasis formed similar clonal patterns. These patterns show, however, that metastasis coincided with a reduction in the ploidy of the parental cancer from hyper-tetraploid to hyper-triploid. Moreover, the karyotype of the metastasis differed from that of the primary cancer in about 27 of an average of 31 metastasis-specific aneusomies (Fig. 14 **a**, **b**, **c** and Table 1). Since the ploidy-shift changed the relative chromosome copy numbers of many aneusomies, the percentage of metastasis-specific aneusomies is, however, larger than if it were based on qualitative differences only (see Table 1). As in all other hyper-diploid cancers, the copy numbers of most non-clonal chromosomes including marker chromosomes differed from clonal averages ± 1 (Fig. 14 **a**, **b** and specifically Fig. 14**c**). Again, the chromosomes with non-clonal copy numbers represent the ongoing karyotypic variation predicted by the inherent variability of cancer-specific aneuploidy (See Background). We conclude that the metastasis IGR-37 is a subspecies of the parental melanoma IGR-39

**Fig. 15:**
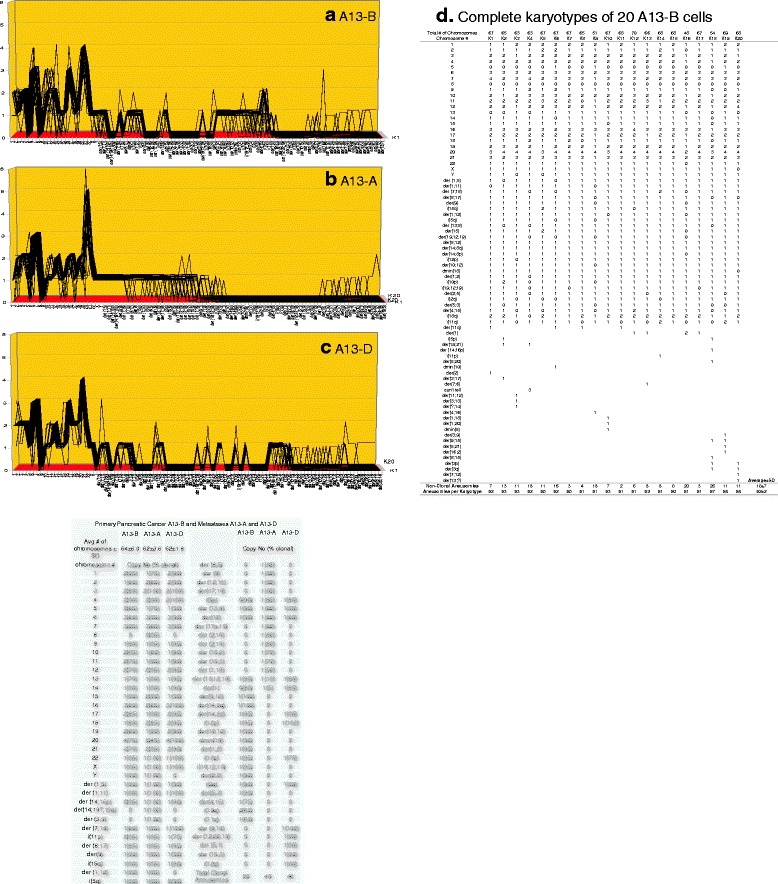
**a**, **b**, **c**, **d** Karyotypic evidence that the metastases A13-A and A13-D are individual subspecies of the pancreatic cancer A-13B. The karyotypic theory of metastasis predicts that metastases have individual clonal karyotypes that differ from those of parental cancers in individual metastasis-specific aneusomies. To test this theory we have compared the karyotype-arrays of the metastases A13-A and A13-D to that of the primary cancer A-3B. The karyotype arrays were again prepared as described for Fig. 9. Figure 15 **a**, **b**, **c** and the attached table show that the chromosomes of the cancer were 55–95% clonal and that of the chromosomes of metastasis A13-A were 75–100% and those of metastasis A13-D were 75–100% clonal, and that all three cancers formed related clonal patterns. As shown in Table 1, the karyotype of the metastasis A13-A differed from that of the primary cancer A13-B in 27 of 49 aneusomies and metastasis A13-D differed from that of the primary in 16 of 49 aneusomies (Fig. 15 **a**, **b**, **c**, **d**). The copy numbers of most non-clonal chromosomes including marker chromosomes differed from clonal averages ± 1 (Fig. 15 **a**, **b**, **c** and specifically Fig. 15**d**). The chromosomes with non-clonal copy numbers represent the ongoing karyotypic variation predicted by the inherent variability of cancer-specific aneuploidy (See Background). We conclude that the metastases A13-A and D are subspecies of the parental pancreatic cancer A13-B

Therefore, we have recently developed a technique, which determines karyotypic clonality based on the clonality of its constituent chromosomes, rather than on the karyotypes as a whole. This technique is able to detect karyotypic clonality, even if a minority of the chromosomes of a given karyotype is non-clonal [76, 108, 109] (Background). The technique arrays the copy numbers of individual chromosomes of 20 cancer karyotypes in 3-dimensional tables. These tables list the numbers of individual chromosomes and of individual marker chromosomes on the x-axis, the copy numbers of the chromosomes and marker chromosomes on the y-axis, and the numbers of karyotypes/metaphases analyzed on the z-axis. The resulting pattern is therefore called a karyotype array. In such arrays, karyotypes with clonal chromosome copy numbers form easily recognizable parallel lines. The arrays shown here also include tables that list the percentages of clonality of each chromosome. The percentages of chromosomal clonality have been calculated from primary data of all chromosomes of all karyotypes analyzed here (Figs. 9c, d; 10c; 11c; 12c, d; 13d,e; 14c and 15d).

As shown in the karyotype arrays of all seven cancers and of the corresponding metastases, the karyotypes of all seven cancers and corresponding metastases were mostly 70–100% clonal (Figs. 9, 10, 11, 12, 13, 14, 15). The 0–30% of non-clonal sets of chromosomes or aneusomies reflects the inherent instability of aneuploidy (Background).

We note that we have counted here all sets of identical chromosomes as aneusomies, including even diploid normal chromosomes of aneuploid cancers. This was justified by the facts that even diploid sets of normal chromosomes are less than 100% diploid in cancers owing to the inherent instability of aneuploid karyotypes (see Figs. 9, 10, 11, 12, 13, 14, 15 and [110]) and that the functions of diploid sets of normal chromosomes are altered in aneuploid karyotypes [111]. In a previous study of metastasis we have termed such aneusomies “chromosomal units” for the same reasons [43].

Table 1 presents a summary of the karyotypic relationships between the seven cancers and corresponding metastases based on shared and individual aneusomies, which is shown below on page 18. The data for this table were derived from Figs. 9, 10, 11, 12, 13, 14, 15.

**Table 1:** Individual and shared clonal aneusomies of metastases and parental cancers

Primary/Metastasis	Total clonal aneusomies	Aneusomies shared with cancer	Metastasis-specific, clonal aneusomies (% of total)
1)Breast, HIM-2 Metastasis, HIM-5	23 23	- 22	- 1 (4%)
2)Melanoma, WM-115 Metastasis, WM-266-4	30 31	- 18	- 13 (42%)
3)Liver, H2P Metastasis, H2M	31 31	- 16	- 15 (48%)
4)Medulloblastoma M-458 Metastasis, M-425	34 28	11	17 (61%)
5)Colon, SW480-C1 Colon SW480-C2 Metastasis, SW620 vs C1 SW620 vs C2	38 39 38 38	- - 13 11	- - 25 (66%) 27 (71%)
6)Melanoma, IGR-39 Metastasis, IGR-37	30 31	- 4	- 27 (87%)^a^
7)Pancreas, A13-B Metastases, A13-A and A13-D	50 49 46	22 30	27 (55%) 16 (35%)

^a^High percentage of individuality reflects ploidy shift (see below, Table 2)

As can be seen in this table, the clonal diversities between cancers and metastases increased with increasing degrees of cancer-specific aneuploidies as follows:

1. The near-diploid breast cancer HIM-2 shared 96% and its clonal aneusomies with the near-diploid metastasis HIM-5.
2. The hyper-diploid melanoma WM-115 shared 60% of its clonal aneusomies with the hyper-diploid metastasis WM-266-4.
3. The hyper-triploid liver cancer H2P shared 52% of its clonal aneusomies with the hyper-triploid metastasis H2M.
4. The hyper-triploid medulloblastoma M-458 shared 32% of its clonal aneusomies with the hyper-triploid metastasis M-425.
5. The hyper-triploid colon cancer SW-480 Clone-1 shared 34% of its clonal aneusomies with the hyper-triploid metastasis or presumed metastasis SW-620 [97–99]. And SW-480 Clone-2 shared 29% of its clonal aneusomies with SW-620.
6. The hyper-tetraploid melanoma IGR-39 shared 13% of its clonal aneusomies with the hyper-triploid metastasis IGR-37. But the size of this numerical discrepancy reflects in part the ploidy-shift of IGR-39 from hyper-tetraploid in the cancer to hyper-triploid in the metastasis.
7. The hypo-triploid pancreatic cancer A13-B shared 44% of its clonal aneusomies with the hypo-triploid metastasis A13-A and 60% of its clonal aneusomies with the hypo-triploid metastasis A13-D, which confirmed and extended a previous study of ours [43].

Regarding the evaluations of the relationships between cancers and metastases based on aneusomies we point out that non-identical numerical aneusomies are typically still related, as for example the trisomy 1 of the cancer H2P versus the tetrasomy 1 in the corresponding metastasis (Fig. 11a). Likewise even structurally distinct aneusomies tend to contain common chromosomal elements. Thus the comparisons based on distinct aneusomies minimize the relationships. This effect is, however, compensated by the juxtaposition of their karyotype arrays, which favor relationships by comparing patterns of all aneusomies as a whole.

In sum all metastases analyzed here confirm the theory that metastases are individual subclones or subspecies of parental cancers, differing from parental cancers in metastasis-specific clonal aneusomies rather than in “elusive” gene mutations [3]. If gene mutations would generate metastases, metastases would have the same karyotypes as parental cancers, but this was not observed. Moreover, if gene mutations were sufficient to confer metastatic phenotypes to cancer, the risk of metastases would be independent of the cancer karyotype. But this was also not observed here as in numerous studies in the past (see Background).

The karyotypic analyses summarized in Table 1 also show the divergence of the karyotypes of metastases from cancers was proportional to degrees of aneuploidy of the parental cancer: The more aneuploid the cancer the more it differs karyotypically from corresponding metastases. This supports the prediction of our theory that cancer-specific aneuploidy catalyzes metastasis by karyotypic variation (Background).

Unexpectedly our comparisons also showed that the total numbers of aneusomies of cancers and of corresponding metastases were consistently very close in all seven pairs of cancers and metastases compared – irrespective of the percentages of aneusomies exchanged in metastasis (Table 1). In view of this conservation of aneusomic complexity between metastases and corresponding cancers, we asked next whether this conservation of aneusomic complexity also applied to the numbers of all constituent chromosomes shared by cancers and metastases and thus to the karyotype as a whole.

### Conservation of the complexity of cancer karyotypes in metastasis

In view of the conservation of cancer-specific aneusomic complexity in metastases (Table 1) we hypothesized that the autonomy of cancers is encoded in the karyotype as a whole and that metastases conserve the specific parental autonomy via the complexity of the parental karyotype. According to this hypothesis cancers acquire new, metastasis-specific host ranges from lateral karyotypic variations between cancers and metastases that do not affect the complexity of the karyotype.

To test the conservation of the complexity of cancer karyotypes in metastases, we compared the average numbers of the chromosomes and the aneusomies of each of the seven cancers to those of the corresponding metastases based on the karyotypic data listed in Figs. 8, 9, 10, 11, 12, 13, 14, 15. The results of these comparisons are summarized in Table 2, which is shown below on page 18. As can be seen in Table 2 the average numbers of all chromosomes, like those of the aneusomies of all seven metastases were almost identical to those of the parental cancers. Even the clonal variabilities of these numbers based on standard deviations were the same. Accordingly each cancer-metastasis pair shared individual ratios of the numbers chromosomes to the numbers of aneusomies.

**Table 2:** Conservation of karyotypic complexity of cancers in metastasis based on their numbers of clonal chromosomes and aneusomies

Cancer-Metastasis pair	Average numbers of chromosomes ± SE	Average numbers of aneusomies ± SE	Ratio chromosomes per aneusomy^a^
1)BrCa HIM-2 Metas HIM-5	48 ± 1 47 ± 1.6	23 ± 0 23 ± 0	2.1 2
2)Mela WM-115 Metas WM-266-4	86 ± 1 82 ± 2	30 ± 1 31 ± 1	2.9 2.7
3)Liver H2P Meta H2M	78 ± 7 86 ± 4	33 ± 1 34 ± 2	2.4 2.5
4)Medullob - 458 Metas- 425	81 ± 2.5 85 ± 2	37 ± 2 28 ± 1	2.2 3.0
5)Colon SW480-C1 SW480-C2 Metas SW-620	53 ± 1 54 ± 2 49 ± 2	40 ± 2 40 ± 1 39 ± 1	1.3 1.4 1.3
6)Mela IGR-39 Metas IGR-37	111 ± 3 73 ± 4^b^	32 ± 1 33 ± 1	3.5 2.2
7)Panc A-13B Metas A-13-A Metas A-13-D	64 ± 6 62 ± 2.6 62 ± 1.8	52 ± 2 51 ± 1 48 ± 2	1.2 1.2 1.3

^a^Numbers of chromosomes divided by numbers of aneusomies generates a form of ploidy; ^b^ploidy shift

There was but one partial exception to this rule that cancer-specific complexity is conserved in metastasis, namely the transformation of melanoma IGR-39 to the metastasis IGR-37, which underwent an apparent ploidy reduction during transformation to metastasis (Table 2). This ploidy shift is indicated here in Table 2 by a decrease in the ratio of chromosome numbers per aneusomies from 3.5 to 2.2. The apparent ploidy shift in metastasis M-425 reflects a loss of parental marker chromosomes and a gain of copy numbers of corresponding intact chromosomes (Fig. 12), thereby maintaining the overall complexity of the cancer in the metastasis.

In view of this we note that karyotypic polyploidization or de-polyploidization does not change the relative proportions of genes within a karyotype and thus not change phenotypes directly. But ploidy variation in cancer cells is typically not exactly even, and is another independent characteristic of cancer cells that may increase or decrease oncogenicity by changing the dosage of genes evenly and non-evenly [76, 77, 112–114]. Its occurrence also seems to be proportional to the degree of cancer-specific aneuploidy [76, 115]. Balanced ploidy changes also occur in the development of some normal animal [116] and plant cells [117] and thus enhance the general output of cells.

In sum our data support the hypothesis that the autonomy of individual cancers is maintained by the karyotype as a whole and is therefore conserved in cancers and corresponding metastases within narrow limits of variation (see Fig. 1, Background). This hypothesis explains prior observations of the conservation of karyotypic complexity in metastasis described by Pearse et al. [88] and us [43, 75].

Conservation of karyotypic and genetic complexity is also observed in conventional Darwinian speciation of mammals, in which new species share with predecessors the same genetic complexity and similar karyotypes [103, 118].

In the following we ask, whether the qualitative karyotypic replacements of multiple cancer-specific aneusomies by metastatic counterparts, shown in Tables 1 and 2, occur simultaneously, in single steps or sequentially in multiple independent steps.

### Are metastases with multiple new aneusomies generated in single or in multiple sequential steps?

The currently prevailing model of carcinogenesis and progressions of cancers holds that normal karyotypes are gradually converted to those of cancer cells - and from cancer cells to variant cancer cells, such as metastases by sequential gene mutations or karyotypic alterations that alter the dosage or structures of certain genes [3, 4, 13, 69, 119–124]. According to this model the numbers of precursors with metastasis-specific aneusomies would decrease with increasing numbers of linearly acquired metastasis-specific aneusomies and thus form the pyramids of intermediates predicted by the step-wise or linear model [3, 4, 69]. To generate from cancers the metastases with multiple metastasis-specific aneusomies described in Table 1, 13 to 27 such steps would be needed, because these metastases differ from parental cancers in 13 to 27 metastasis-specific aneusomies. If so many precursors do indeed exist, they should show up among the many variants of parental cancer karyotypes before or at metastasis. But no such hypothetical karyotypic precursors were found in our karyotype arrays of the six cancers that spawned metastases with multiple aneusomies (Table 1). This conclusion is based on complete analyses of all aneusomies of 20 karyotypes of the six cancers from which metastases with multiple new aneusomies arose (and even of corresponding metastases) that are shown in Figs. 10c, 11c, 12c, 13d and e, 14c and 15d.

This result can, however, be explained by an alternative model, which holds that all chromosomal alterations setting apart cancers from normal cells, and likewise all chromosome alterations setting apart metastases from cancers occur simultaneously in a single step. This proposal is based on the absence of the many intermediates or precursors predicted by the linear model, which are probably absent, because they are not viable [43, 76, 77, 125–127] and the finding of others that all aneusomies of the cancers analyzed carried genetic markers only from one, instead of both sets of normal parental chromosomes of an organism [128, 129].

This single-step model predicts that (1) there would be no stable precursors of metastases with less than the full set of authentic metastasis-specific aneusomies, and that (2) all metastases retain the karyotypic complexity of parental cancer as a whole, despite metastasis-specific aneusomic variations (see previous section). At the same time the single-step model also predicts there would be no precursors of the many non-clonal, non-metastatic variants of cancer-karyotypes with multiple non-parental aneusomies (See Figs. 8, 9, 10, 11, 12, 13, 14, 15).

As pointed out above and summarized in Table 2 our karyotypic analyses shown in Figs. 10, 11, 12, 13, 14, 15 exactly confirm these predictions of the single-step model: (1) There were no intermediates of metastases with subsets of the aneusomies of authentic metastases in Figs. 10, 11, 12, 13, 14, 15; and (2) The karyotypic complexity of metastases with multiple non-parental aneusomies was practically the same as that of parental cancers (Table 2).

This absence of precursors of metastases confirms previous results from others including us [43, 76, 77, 126, 127]. In addition our results confirm the predicted absence of precursors of the many non-metastatic variants with multiple aneusomies and with the same complexities as their metastatic counterparts (Figs. 10, 11, 12, 13, 14, 15). In view of this we conclude that our data support the single-step model of metastasis.

In agreement with the single-step model we found, unexpectedly, one specific cell, in which the whole karyotype of the metastatic medulloblastoma M-425 with all of its 24 individual aneusomies was already or was still present in the primary cancer, M-458 (marked yellow in Fig. 12c). This result argues against the sequential theory, because predictably more precursors with less than the 24 new metastasis-specific aneusomies of M-425 should have been present in this cancer than the 1 in 20 with all 24 M-425-specific aneusomies we found. But this was not the case.

The simplest explanation for this result suggests that metastasis is a stochastic single-step variation of the parental cancer karyotype, which expands the host-range of the parental clone to non-native tissues and thus favors metastasis while retaining its native host range. So this M-425 ‘metastasis’ would have continued to replicate in the primary cancer after a sibling had metastasized to a new site. Thus, this observation lends further support to our theory that metastases are single-step karyotypic variants of clonal cancer karyotypes.

By contrast generation of metastases by independent sequential karyotypic variations of precursors, would predict metastases with unpredictable complexities that would not necessarily match those of parental cancers. In addition the sequential model would predict intermediates with aneusomies shared with authentic metastases in M-458, which we did not observe, as shown by the complete karyotypic analyses of M-458 in Fig. 12c.

### Is metastasis a model for Darwinian speciation?

Our results on the karyotypic evolution of metastatic subspecies from cancers may also serve as an experimental model for theories that karyotypic alterations, rather than gene mutations [130], have generated new conventional species such as those proposed by Goldschmidt (1940) [131], White (1978) [118], King (1993) [132], Vincent (2011) [90] and Heng (2015) [133].

In contrast to these karyotypic theories of speciation, the evolution of Darwinian species is widely presented as a stepwise accumulations of genetic mutations [130], much like the currently established models of carcinogenesis [3, 4]. But testable mutations of this “Neo-Darwinian theory” have not been described [103, 130]. Moreover, the Neo-Darwinian theory does not explain the karyotypic individuality of all species [103] and of all cancers [75]. Instead, mutations predict common targets on common karyotypes rather than karyotypic individuality [90, 134].

Thus our results on the origins of metastases from cancers offer a testable karyotypic model for the origins of conventional species, especially the mammals, from their immediate precursors [103, 118].

## Conclusions

The theory that cancer-specific aneuploidy catalyzes the karyotypic evolution of metastases from cancers, explains the following characteristics of metastasis, which are not predictable by the competing mutation theory:

1. The dependence of metastasis on the degree of cancer-specific aneuploidy, because aneuploidy catalyzes karyotypic variations from which metastases arise at aneuploidy-dependent rates: the more aneuploid the cancer the higher its proclivity to metastasize. This explains the early studies summarized in the Background that have linked the risk of metastasis to the degrees of cancer-specific aneuploidy. Catalysis by cancer-specific aneuploidy further suggests that the “mutant alleles” thought to “preordain” or “pre-determine” the proclivity of cancers to metastasize [69, 74] are instead cancer-specific aneuploidies.
2. The individuality of metastases as results of unpredictable stochastic karyotypic variations that convert cancers to metastases - much like random karyotypic variantions explain the individuality of conventional species.
3. The conservation of the complexities of cancer karyotypes in metastasis - as in conventional speciation [103].
4. The “abrupt” origins” and the “unpredictable” and “independent” phenotypes of cancers in metastasis, known as Foulds rules of progression [5], by the low probability and phenotypic unpredictability of new subspecies generated by random chromosomal rearrangements - much like the abrupt origins of new species in conventional speciation.

## Methods

### Origins of seven cancer cells and corresponding metastases

(1) A near-diploid breast cancer, termed HIM-2 and a brain metastasis, termed HIM-5 were a generous gift of Elaine Mardis. They were isolated and sequenced as described by her and her collaborators in 2010 [16]. (2) A hyper-triploid primary melanoma WM-115 and a metastasis of undefined origin, termed WM-266-4 [21–23] were obtained from American Type Culture Collection. (3) A liver cancer termed H2P and a metastasis in the portal vein to the liver termed H2M was isolated and provided by Xin-Yuan Guan [35]. (4) A hyper-triploid medulloblastoma termed Med-458 and a metastasis from a cerebrospinal fluid relapse termed Med-425 [95, 96] were kindly transferred to us by the senior author of this study, Darell D. Bigner (personal communication, 2014). (5) A hyper-diploid colon cancer SW-480 and a lymph node metastasis SW-620 [97–101] with evidence shown above that both are metastases from an unknown common precursor were obtained from two sources, SW-480 was a purchase from American Type Culture Collection and SW-620 was a kind gift from Josh Nicholson (Virginia Polytechnic Institute and State University, Blacksburg, Virginia). (6) A hyper-tetraploid melanoma from the leg, termed IGR-39 and a hyper-triploid metastasis from the groin of young male patient, termed IGR-37 [102] were purchased from DSMZ-Deutsche Sammlung von Mikroorganismen und Zellkulturen GmbH, Braunschweig, Germany. (7) A hypo-triploid pancreatic cancer termed A13-B and two independent hypo-triploid metastases, a pancreatic metastasis A13-A and a liver metastasis A13-D, were obtained as described previously [43]. All cultures were grown in RPMI 1640 medium (Sigma Co.) supplemented with 3 to 5% fetal calf serum and antibiotics as described previously [101, 102].

## Karyotype analyses

One to two days before karyotyping, cells were seeded at about 50% confluence in a 5-cm culture dish with 3 ml of the medium described above. After reaching ~75% confluence, 250–300 ng colcemid in 25–30 μl solution (KaryoMax, Gibco) was added to 3 ml medium. The culture was then incubated at 37 °C for 4–8 h. Subsequently cells were washed once with 3 ml of physiological saline, dissociated with trypsin, pelleted and then incubated in 0.075 molar KCl at 37 °C for 15 min. The cell suspension was then cooled in ice-water, mixed (‘prefixed’) with 0.1 volume of the freshly mixed glacial acetic acid-methanol (1:3, vol. per vol.) and centrifuged at 800 g for 6 min at room temperature. The cell pellet was then suspended in about 100 μl supernatant and mixed drop-wise with 5 ml of the ice-cold acetic acid-methanol solution and then incubated at room temperature for 15–30 min or overnight at -20C. This cell suspension was then pelleted and was then either once more re-suspended in fixative and pelleted, or was directly re-suspended in a small volume of the acetic acid-methanol solution for microscopic examination. For this purpose an aliquot of a visually turbid suspension was transferred with a micropipette tip to a glass microscope slide, allowed to evaporate at room temperature and inspected under the microscope at x 200 for an adequate, non-overlapping density of metaphase chromosomes. Metaphase chromosomes attached to glass slides were then hybridized to color-coded, chromosome-specific DNA probes as described by the manufacturer, MetaSystems (Newton, MA 02458). Karyotypes were analyzed under a fluorescence microscope, as described by us previously [17, 49].
